# Realizing the potential of mobile interventions for education

**DOI:** 10.1038/s41539-024-00289-9

**Published:** 2024-12-12

**Authors:** Jasmin Breitwieser, Andreas B. Neubauer, Florian Schmiedek, Garvin Brod

**Affiliations:** 1 Education and Human Development, DIPF | Leibniz Institute for Research and Information in Education, Frankfurt am Main, Germany; 2 IDeA Center for Research on Individual Development and Adaptive Education of Children at Risk, Frankfurt am Main, Germany; 3 Institute of Psychology, RWTH Aachen University, Aachen, Germany; 4 Institute of Psychology, Goethe University Frankfurt, Frankfurt am Main, Germany

**Keywords:** Human behaviour, Psychology

## Abstract

Mobile devices are ubiquitous, but their potential for adaptive educational interventions remains largely untapped. We identify three key promises of mobile interventions for educational research and practice: 1) intervening when it is most beneficial (i.e., “just-in-time adaptivity”), 2) estimating causal effects of interventions in ecologically valid settings, 3) considering the impact of context on the effectiveness of interventions. We discuss the challenges and next steps to advance this field.

## Realizing the potential of mobile interventions for education

What to do when a student can’t bring herself to study regularly and always crams right before the exam? What to do when a student feels stressed or anxious thinking about the next day at school? What to do when a student can’t focus on his homework? These questions are perennial issues in educational psychology. Especially when there is no teacher around to intervene, students are left on their own to cope with the situation. Can mobile devices help?

Learning is increasingly taking place outside formal learning settings, largely due to the widespread availability of mobile devices. Mobile devices offer students the flexibility to access content anywhere and anytime^1^. However, many of the most persistent self-regulatory challenges students face are exacerbated in out-of-school contexts where teacher support is not readily available. These self-regulatory challenges include recognizing good opportunities to study, motivating oneself to study, coping with emotional distress, and resisting distractions. Although the idea that educational interventions are most effective when provided “just in time” is not new^2,3^, its application needs to be extended beyond specific (digital and non-digital) learning environments to moments in everyday life. We argue that by collecting various types of data about a student’s internal state and environmental factors, mobile devices are able to flexibly adapt interventions to these changing contexts. Thus, they can provide the right type and/or amount of support when it promises to have the greatest impact, helping students cope with situations like those described above.

In exploiting the full potential of mobile devices for research and practice, education has the opportunity to learn from the field of health psychology, where mobile interventions are rapidly gaining prominence. We see vast potential in applying mobile interventions and related experimental designs to support students and to advance our understanding of educational processes. In describing these promises, we draw on examples from mobile health (mHealth) that we believe are illustrative for education. We also discuss early attempts to apply mobile interventions to education, thereby illustrating their versatility and potential. We conclude by highlighting key challenges of using mobile interventions in education and provide recommendations for future research that we hope this paper will stimulate.

## What are mobile interventions?

The rapid proliferation of mobile devices among students has sparked discussions about their role in education. A recent special issue of *Contemporary Educational Psychology* highlighted two dominant research traditions in this area^4^. The first, m-learning, focuses on the use of mobile technologies to deliver academic content, thereby enabling “learning across multiple contexts, through social and content interactions” (ref. ^1^, p. 4). Typical research topics include user experiences and user engagement with learning apps^5–7^ (but see ref. ^8^ for a critique). M-learning thus considers mobile devices primarily as learning tools.

The second approach uses mobile technology to collect data on psychological processes and contextual factors to gain insights into psychological theories of learning. For instance, experience sampling combined with location tracking or physiological measures can probe the contextual nature of student engagement^9,10^. Unlike m-learning, this approach is not limited to learning experiences on mobile devices. Research on topics such as achievement emotions or value-control appraisals uses mobile devices to collect subjective experiences in a variety of formal and informal, digital and analog learning settings, which has paved the way for advances in emotional and motivational theories^11^. In essence, this highlights the potential of mobile devices as research tools.

In this article, we propose *mobile interventions* as a third approach to harness the potential of mobile technology for education. Mobile interventions are not a competing concept, but should rather be seen as a complement or extension of the aforementioned approaches. Unlike m-learning, they do not refer to the delivery of educational content on mobile devices, but to interventions that aim to change students’ behavior, experiences, or cognitive processes relevant to learning - whether in analog or digital, formal or informal learning contexts. They are usually delivered via mobile devices to take advantage of unique affordances that cannot be easily replicated by other methods. First, they enable the repeated delivery of intervention prompts in different contexts and at shorter time intervals (e.g., multiple times per day). The focus is thus on changing psychological states—with the understanding that repeated changes in states can lead to longer-lasting changes in skills or behavior^12–14^. Second, mobile devices facilitate the collection of high-resolution data on students’ internal and external states alongside the intervention, which can be used to explain, predict, and enhance the effectiveness of the intervention in these changing contexts. Advances in other educational technologies, particularly intelligent tutoring systems, show how sophisticated systems can use multimodal data to provide just-in-time support that promotes students’ self-regulated learning skills^15^. Mobile interventions go beyond these systems by providing just-in-time support outside regular learning environments. Therefore, they allow to address educational challenges that occur not when students are already learning, but before or between learning sessions, such as anticipatory negative emotions, procrastination, or recognizing a good opportunity to start learning.

In the remainder of this article, we provide several examples that illustrate how mobile interventions and experimental designs that are aimed at answering scientific questions about mobile interventions can serve a wide variety of purposes for educational practice and research. However, we suggest that it is these two features – the repeated delivery of intervention prompts at shorter time intervals and the utilization of high-resolution data collected alongside the intervention – that set mobile interventions apart from more traditional or analog forms of intervention.

## What is the promise of mobile interventions in education?

In the following, we will describe what we believe are the key promises of mobile interventions and related experimental designs (specifically micro-randomized trials) in education: 1) being able to intervene just in time, 2) estimating causal effects of interventions in ecologically valid settings, 3) taking into account the impact of context on the effectiveness of interventions.

### Being able to intervene just in time

The effectiveness of an intervention often hinges on its timing. A student receiving a study reminder during a social event is likely to ignore it, whereas if she receives the same reminder while waiting for the bus, she may be more inclined to engage with it. Similarly, a student feeling particularly anxious one night might benefit from a prompt to perform a breathing exercise, while on other nights, a prompt to journal might reinforce his sense of well-being. These examples illustrate the motivation for just-in-time adaptive interventions (JITAIs) in education, that is, providing support precisely when and where it is most beneficial^16^.

Mobile devices are ideally suited to implement and adapt interventions just in time, especially when teachers or other human support is not readily available. Smartphones and -watches can collect various types of data when they are actively used (e.g., screen time, app usage), passively worn (e.g., location tracking, electrodermal activity), or record students’ reports on their current behavior, affect or thoughts (i.e., experience-sampling). Drawing on the collected data, an algorithm can provide different types and/or amounts of prompts based on decisions made in real time (e.g., sending study reminders based on location data, prompting deep abdominal breathing or journaling based on electrodermal activity). Therefore, mobile devices make it possible to flexibly adapt an intervention to a person’s changing internal and contextual state in any everyday situation.

JITAIs have gained much traction in health research and practice^16^. A notable example is the HeartSteps program, which integrates an app with a Fitbit activity tracker to promote physical activity^17–20^. The app sends messages that prompt a specific behavior (e.g., taking a walk) based on reinforcement learning algorithms that predict how likely a person is to respond to a prompt in different contexts (e.g., weather conditions, location) and adjust future delivery accordingly^19,21^. In addition to dose, HeartSteps also adjusts the content of prompts. This was based on the results of a micro-randomized trial in which, each day with a certain probability, the app sent a motivational message that varied in its focus, which enabled research into the contextual effectiveness of the messages^20^. We argue that the intensive, iterative research program on which HeartSteps is based can serve as a model for mobile intervention research in education.

**Fig. 1:**
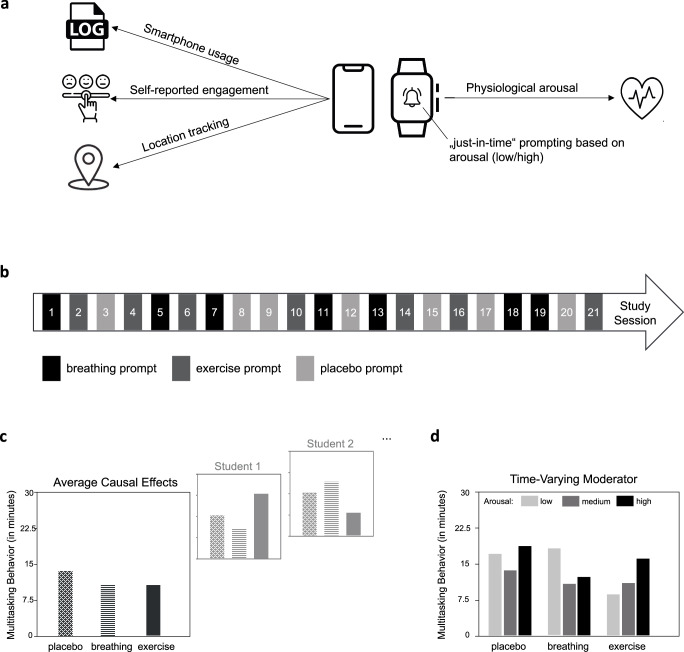
Schematic Illustration of a Hypothetical Mobile Intervention. **a** A mobile intervention system consisting of a mobile app and a smartwatch that tracks various types of data and presents “just in time” prompts to regulate arousal levels with the goal of reducing multitasking behavior. **b** A micro-randomized trial to test the causal effects of three different types of prompts on multitasking behavior; each study session, students are randomized to one of the three conditions. **c** Hypothetical results of the micro-randomized trial showing the average effectiveness of the different prompt types on multitasking behavior (the average causal effects are the contrasts between the conditions); students differ in how the prompts affect their behavior (between-person differences in within-person causal effects). **d** The arousal level at the time of prompting influences the effectiveness of the different prompt types as a time-varying moderator.

JITAIs are highly promising when applied to educational contexts as well, with applications as diverse as in mHealth. Fig. 1 presents a hypothetical mobile intervention designed to reduce media multitasking during studying. Models of media multitasking have suggested that individuals may multitask to regulate their arousal levels^22^. Therefore, combining a mobile app and a wearable device, akin to HeartSteps, could offer promising avenues for just-in-time intervention. The system could monitor physiological arousal via electrodermal activity and track multitasking behavior via smartphone tracking and/or experience sampling. If arousal levels are detected as unusually high or low, the system could suggest activities, like breathing exercises or physical movement, to regulate them (see Fig. 1a). Thus, the intervention would adapt to students’ changing states to prevent unfavorable study behaviors. Harnessing the power of mobile technology in this and similar ways could revolutionize individualized educational interventions.

### Estimating causal effects of interventions

Using mobile devices to repeatedly assess students’ current affect or thoughts (i.e., experience sampling) has become a popular tool in educational research. This method provides insights into otherwise inaccessible settings and experiences close to when they are happening^23^, and has helped uncover within-person couplings between students’ social, cognitive, and affective states and their performance and well-being in and out of school (e.g., refs. ^10,24–28^). A major advantage is that these couplings control for all (including unobserved) time-*invariant* confounders. Nevertheless, causal inference is still quite limited by the fact that they cannot control for all potential time-*varying* confounders (see ref. ^29^). For instance, the intraindividual link between arousal and media multitasking from our earlier example has been shown at a correlational level^30^, leaving open the possibility that the intention to engage in rewarding distraction could be the reason for heightened arousal prior to the multitasking event, rather than multitasking being triggered by a change in arousal. A powerful experimental design enabled by mobile devices allows the testing of causal effects using micro-randomization.

Micro-randomization involves the repeated random assignment of students to receive or not receive an intervention or a particular variant of an intervention^17,31^. In contrast to a traditional randomized controlled trial, in a micro-randomized trial randomization is performed within individuals over time (i.e., intraindividual randomization), such that, in a trial lasting several weeks, each person may be randomized tens or hundreds of times. This can be viewed as an evolution of the single-case design, which has a long tradition in educational research, featuring repeated alternations between one or two treatment conditions and a baseline condition in a single participant^32^. For example, in a micro-randomized trial with many students over several weeks, an app could send prompts during students’ study sessions, randomly encouraging them to engage in either physical activity or breathing techniques (Fig. 1b). By comparing multitasking behavior after these prompts with behavior after placebo prompts within students and then integrating these results across students, micro-randomization allows researchers to test the average causal effect of the prompts on behavior at the individual level.

Micro-randomization has clear advantages over randomized controlled trials. First, it increases statistical power. Repeated randomization within individuals increases the number of data points per person, thus allowing researchers to draw more firm conclusions compared to a standard randomized controlled trial with the same sample size. Second, and most importantly, micro-randomization allows researchers to estimate the heterogeneity in within-person causal effects^29,33^. Even if an average causal effect of the prompt on media multitasking could be identified, this effect likely varies considerably between students (Fig. 1c). For example, some students may be more prone to multitask when arousal levels increase after exercise, while others may be more likely to multitask when arousal levels decrease after a breathing exercise. Thus, micro-randomized trials provide an ideal experimental design for developing and optimizing JITAIs based on the proximal causal effects of intervention options^31^. However, for findings from micro-randomized trials to pave the way for personalized interventions, it is important to determine to what extent the variability in causal effects is systematic and, ideally, what factors explain it. Fortunately, mobile technology may be able to help in this regard as well.

### Taking into account the impact of context on the effectiveness of interventions

One of the most pertinent issues of educational research is that of personalization: which interventions and instructions work best for which students. Historically, the focus has been on students’ abilities and traits, as exemplified by research on aptitude-treatment interactions^34^. This can be applied to mobile intervention research as well by asking which student characteristics explain the heterogeneity of causal effects revealed by micro-randomized trials, and personalizing the intervention accordingly. In mHealth research, hybrid experimental designs have been developed that can help identify which students respond strongly to the intervention and which do not, and subsequently adapt the intervention (for an overview, see ref. ^35^). These adaptations are typically implemented on slower timescales (weeks/months) and refer to human-delivered intervention components, although this principle could be applied to mobile-delivered intervention components as well. For instance, it may take several weeks to reliably determine that a student is not responding desirably to physical activity prompts (i.e., a certain efficacy criterion is not met) and would then be switched to breathing prompts, or vice versa. Thus, personalization can be achieved by focusing on students’ responses to the intervention itself, rather than having to attribute these responses to specific student characteristics.

Beyond stable student characteristics, however, micro-randomized trials offer the unique opportunity to understand which intervention components and instructions work best for which students *at what time*^36^. In more technical terms, they enable the investigation of time-varying contexts as moderators of intervention effects. Applied to education, this means that the effects of interventions can be tested in various educational contexts, both in and out of school (e.g., first vs. last lesson of the day, chess vs. sports club). In addition to the external context, interventions can vary in effectiveness depending on students’ internal states that fluctuate over time (e.g., mood, motivation). For instance, a student’s arousal level at the time of receiving a prompt might influence the effectiveness of physical activity or breathing prompts on multitasking behavior (Fig. 1d). So far, these context effects have been difficult to test because the temporal resolution of interventions and assessment has been too coarse. Using the various data collected by mobile devices, micro-randomized trials allow researchers to explicitly test the effects of time-varying moderators of intervention effectiveness. This cannot only provide a solid basis for the development of JITAIs but could also contribute to a better understanding of the psychological mechanisms involved.

## Initial examples of mobile interventions in education

In the previous sections, we illustrated the promise of mobile interventions in education using the hypothetical example of an advanced JITAI that uses both a mobile app and a wearable device to reduce students’ media multitasking. In this section, we turn to existing mobile intervention studies that we have conducted in our own labs. In addition to providing more examples of potential use cases for mobile interventions in education, we hope to offer insights into the early stages of such research programs.

### PROMPT

PROMPT is a mobile app designed to improve the self-regulated learning skills of 9–13-year-olds. The app includes a mobile intervention that specifically targets two important strategies: the learning strategy of distributing study sessions over time^37^ and the metacognitive strategy of planning when and where to study^38^. Videos integrated into the app explain the two strategies and help children formulate a plan for themselves. Most relevant to the current paper, the app also periodically sends a prompt reminding children of the benefits of distributed practice and their study plan.

**Fig. 2:**
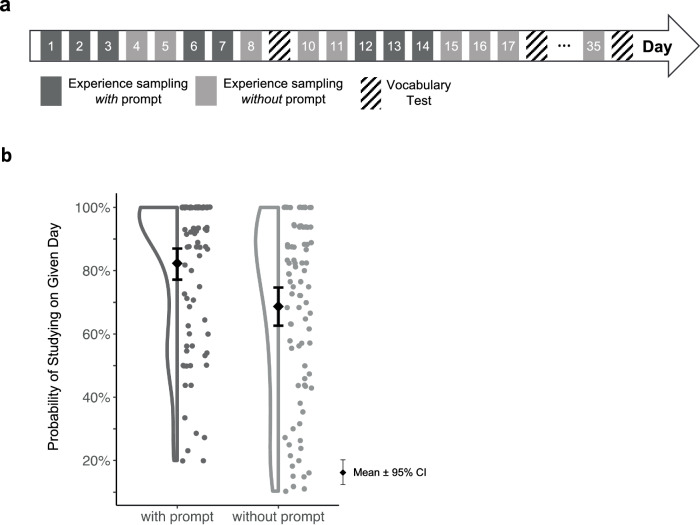
Illustration of the PROMPT Study. **a** Schematic overview of the micro-randomized trial design. **b** Results showed that children were more likely to study vocabulary on days with prompt than on days without prompt.

To evaluate the effectiveness of this intervention, we conducted an intensive longitudinal study in which 130 children used both the PROMPT app and a separate vocabulary app (see ref. ^39^). The goal was to help children use the vocabulary app more regularly. Over the course of 36 days, the children used the PROMPT app daily to answer questions related to their learning (experience sampling). On some days, they also received the reminder prompt at the end of the questionnaire (Fig. 2a). Our results showed that children were more likely to study with the vocabulary app on days with a prompt compared to days without prompt (Fig. 2b). These findings suggest that the prompt had a causal effect on study behavior.

Importantly, the combination of the micro-randomized trial with experience sampling data, along with logfile data from both apps, allows us to investigate potential moderators of the prompting effect. For instance, a certain level of study motivation may be required for the prompts to be effective. Results from our latest research suggest that planning prompts are only effective if students create plans that are of high quality, and that the quality of these plans tends to increase when students report higher motivation to study the next day^40^. Building on these findings, we are now working towards a system that integrates personalized feedback generated by large language models (LLMs) to help students create higher-quality plans. In addition, the findings suggest that adapting the intervention based on students’ motivation could be a promising direction for developing a JITAI.

### UPWIND

Results from observational experience sampling studies suggest links of children’s well-being and cognitive performance with various behaviors and experiences in daily life. For instance, sleep duration and sleep quality were associated with well-being^26,41^ and working-memory performance^25^ in school children’s daily lives. Perseverative thoughts such as worry and rumination^42^ or peer interactions in school^43^ were also identified as correlates of day-to-day fluctuations in children’s well-being. Additionally, children differed in the size, but also direction of these within-person associations. These patterns of results suggest that interventions targeting sleep, perseverative cognitions or other presumed antecedents of well-being and cognitive performance in children’s daily lives might have beneficial outcomes – yet different interventions might be needed for different children.

**Fig. 3:**
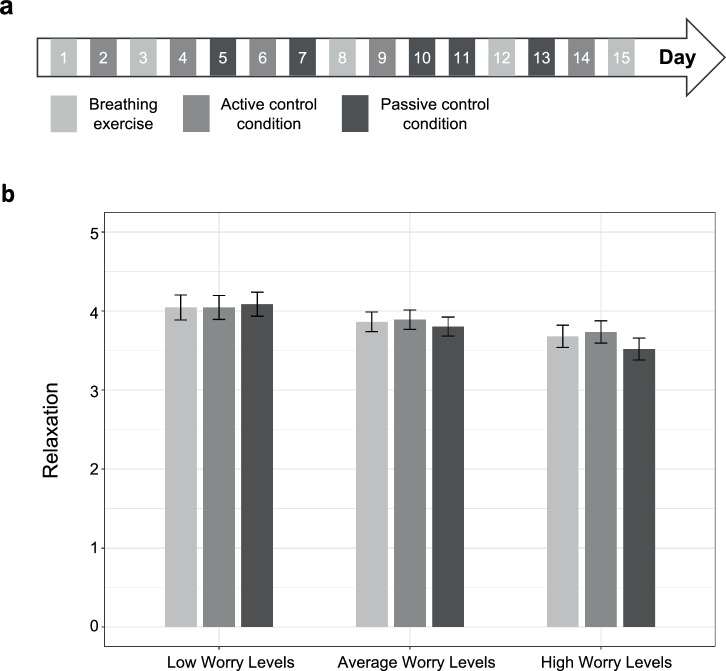
Illustration of the UPWIND study. **a** Schematic overview of the micro-randomized trial design. **b** Results showed that the breathing intervention increased relaxation on days when children worried more than usual.

The UPWIND project was intended to test these ideas by implementing interventions in school children’s everyday lives and examining heterogeneity in the effectiveness of the interventions between children. We have so far tested the effectiveness of a slow-paced diaphragmatic breathing intervention to reduce negative affect and increase relaxation in children’s daily lives^44^. In this study, 171 children between 9 and 13 years took part in a micro-randomized trial. On each of the 15 study days, children were assigned to one of three conditions: (a) to perform a video guided, slow-paced diaphragmatic breathing exercise for three minutes; (b) an active control condition (watching an animated education video); or (c) a passive control condition (no video; Fig. 3a). There was no main effect of the breathing exercise on either negative affect or relaxation, but preregistered moderation analyses suggested that the breathing exercise increased relaxation on days when children worried more than usual (Fig. 3b). This pattern shows that the effectiveness of the intervention depends on context factors, further highlighting the need for explicitly considering these factors in the implementation of mobile interventions.

To further examine differential effects of different intervention approaches, we are currently conducting a micro-randomized trial in school children, who receive both the diaphragmatic breathing exercise at home and a physical activity in the school setting. Combining several intervention components in the same sample will allow a more detailed account of between-person differences in the effectiveness of each component: Do children who profit more from the breathing exercise also profit more from the physical activity break? Or do some children who profit from the more activating physical activity break benefit less from the more relaxing breathing exercise?

## Challenges and perspectives for future research

Mobile interventions have great potential to improve student learning. However, their implementation for educational purposes presents distinct challenges. In the following, we address five key areas: (1) balancing just-in-time support and student agency, (2) potential negative side effects of mobile interventions, (3) engagement in mobile interventions, (4) methodological challenges, and (5) ensuring data protection.

First, there is an inherent tension between providing on-demand support and promoting student agency. While mobile interventions can provide support whenever needed, they run the risk of reducing student motivation and undermining the development of self-regulated learning skills. This issue has been discussed before^45–47^ but may be exacerbated in mobile interventions that could intrude on critical moments that would otherwise serve as opportunities for students to practice their capacity to act independently. Recent research proposed the idea of “adaptive assignment of agency levels to learners” (ref. ^45^, p. 12), which implies that JITAIs should adapt based on student progress, offering less support as students become more advanced. Moreover, mobile interventions should prompt students to apply self-regulation strategies on their own, rather than simply reminding them to act in a certain way. For example, prompting students to create their own individual study plans helped students maintain a regular study routine (Blinded)^39^, whereas simple reminders to study more often created an over-reliance on the external prompts (Blinded)^48^. Future research could explore how artificial intelligence, particularly LLMs, could be leveraged to adaptively suggest activities that promote self-generative and self-regulated activities or provide personalized feedback (see ref. ^46^).

Second, while mobile interventions have great potential to improve education, it is important to remain cautious about potential negative side effects. Concerns about media multitasking^49^, decreased attention^50^, and excessive screen time^51^ are well documented, and mobile interventions could inadvertently aggravate these challenges. Mobile prompts could be a gateway to using other apps that are just one click away, thus increasing screen time. Future research should examine whether certain learners experience more negative effects from mobile interventions and how these effects could be mitigated, thus focusing not only on intended, but also on unintended outcomes. Solutions could include combining mobile interventions with digital self-control tools^52^, such as screen time limits. However, mobile interventions could also be a distraction in themselves if prompts disrupt learning activities. This further underscores the importance of optimal intervention timing, which includes identifying *suboptimal* time points at which a student may be more susceptible to the potential distraction of a mobile prompt^16^.

Third, no mobile intervention can be effective if students do not engage with it. Engagement is thus a critical issue discussed in the mobile intervention literature^53^. The Technology Acceptance Model^54^ provides a framework for the factors that determine engagement, with perceived usefulness and ease of use as the most proximal ones. In educational settings with younger students as the target group, the focus should also be on fostering children’s enjoyment of engaging with an app^55^. Qualitative methods, such as participatory design approaches, can be used to collaborate with learners on a design that fosters engagement^56^ and to obtain rich feedback. However, we caution against a technocratic approach that seeks to solve the challenge of engagement through technological solutions alone. Even when learning takes place outside formal classroom settings, it is still embedded in a broader social context. Therefore, research should focus on how support from teachers, parents, or peers can be effectively integrated into mobile intervention programs, for instance by integrating reflections about the mobile intervention into lessons, helping with technical difficulties, and more. Research in mHealth shows that interventions are more effective with human support^57,58^. Taken together, we suggest combining technological and human-level factors to promote engagement.

Fourth, developing and evaluating mobile interventions poses several methodological challenges. One major issue is adherence to prompts in micro-randomized trials. Despite best efforts to boost engagement, some participants will not engage in a behavior when prompted, or engage without being prompted. In many mHealth studies, this is manageable because the targeted behavior serves as both treatment and outcome (e.g., achieving a daily physical activity level). The causal effect of the intervention on the behavior can then be estimated directly, while effects on distal outcomes (e.g., body weight) can be estimated using traditional (e.g., pretest-posttest) designs. In education, however, we are often interested in the effect of a particular behavior (e.g., performing a breathing relaxation technique) on a different outcome (e.g., reduced arousal, improved learning). Here, adherence issues become more problematic. Estimating the effect of the randomized prompts on the outcome regardless of whether or not the behavior was performed in the (non-)prompted situations (analogous to an intent-to-treat analysis in medical trials) may be the most relevant effect from a practical perspective. From a research perspective, however, (individual differences in) the effect of the behavior on the outcome is often of interest. Here, the use of instrumental variable estimation methods can allow estimation of the effect of interest in the case of non-perfect adherence^59^. The applicability and usefulness of these approaches in different applications of micro-randomized trials still remains to be thoroughly investigated.

Another methodological challenge is whether the intervention can be considered to have an “on/off” characteristic, i.e., relatively immediate but also transient and (at least partially) reversible effects. In this case, micro-randomized trials are useful because the repeated on/off switching of the intervention can causally induce variability in the outcome variable, allowing estimation of average and individually differing causal effects. For example, relaxation techniques often have temporary effects that suit this design. However, if interventions have longer-lasting effects, micro-randomized trials become less applicable. In addition, the reversibility of certain behaviors may be questionable, for example in apps that encourage learners to use certain strategies. If learners adopt the strategy into their behavioral repertoire, the control over the behavior provided by the randomized prompts may be lost, thus reducing the possibility to investigate (individual differences in) certain phenomena. In the extant research on single case (e.g., ABAB) designs, such effects and the resulting methodological limitations have been known and discussed for quite some time (ref. ^60^, pp. 138-139; ref. ^32^, pp.128-129). One solution could be to include additional groups in the design to control for changes in baseline levels. For instance, in the PROMPT study, we included a group that used the app but without receiving prompts. This allowed us to compare the study behavior of this control group with the study behavior of the intervention group on days when the intervention group did not receive prompts to see if the effects of prompting carried over to days without prompts^39^. Which variations of the prototypical within-person micro-randomized trial design are useful for which research questions could be a valuable direction for future research.

Fifth, the field must address issues regarding data protection and privacy. Advanced JITAIs may require extensive data collection^61,62^, including sensitive information such as location tracking^63^. Furthermore, integrating LLMs like ChatGPT —or any third-party service—into a mobile intervention raises concerns about data privacy and user consent. In educational applications, unique challenges arise due to the involvement of minors, who require additional protections under regulations like COPPA in the U.S. or GDPR in Europe. Parental consent becomes essential, but this adds complexity in terms of managing access. It is therefore important to develop best practices that integrate lessons from mHealth^64,65^, but also consider the unique conditions of educational applications, such as the young age of participants and the involvement of parents and teachers.

## Conclusion

With this review, we hope to contribute to kickstarting a new field of mobile intervention research in education, comparable to the emergence of mHealth research in the health sciences. We have highlighted three key promises that could greatly enhance educational practice and research: first, the ability to intervene when and where it is most beneficial (i.e., ‘just in time’), thereby enhancing the effectiveness of interventions; second, the possibility of estimating (individual differences in) causal effects of interventions in ecologically valid settings; third, the ability to take into account contextual influences measured by mobile devices, thus allowing a more precise understanding and prediction of intervention effectiveness based on time-varying moderators. However, it is important to acknowledge that moving mobile interventions research into the educational domain is not a mere replication of mHealth research strategies. As we have argued, it presents both substantive and methodological challenges that offer ample opportunities for future research. While mHealth strategies serve as a useful blueprint, education presents a unique set of conditions that will require careful consideration and innovative solutions.
